# High-quality ^13^C-detected structural analysis of mass-limited amyloid samples using a CPMAS CryoProbe and moderate magnetic fields

**DOI:** 10.1016/j.ssnmr.2025.102028

**Published:** 2025-10

**Authors:** Sara Andrés-Campos, Gustavo A. Titaux-Delgado, Fátima C. Escobedo-González, Miguel Mompeán

**Affiliations:** 1 Instituto de Química Física Blas Cabrera, Consejo Superior de Investigaciones Científicas (IQF-CSIC), Serrano 119, 28006 Madrid, Spain

## Abstract

Solid-state NMR (SSNMR) of biomolecules typically requires several milligrams of sample to achieve sufficient sensitivity for multidimensional experiments, especially when relying on ^13^C detection. Recent developments in fast magic-angle spinning (MAS) and ^1^H-detected methods have enabled the use of submilligram samples in reduced-diameter rotors, but these approaches demand advanced hardware and often suffer from limited ^1^H chemical shift dispersion. Here, we demonstrate that a CPMAS CryoProbe enables the acquisition of high-quality ^13^C-detected 2D and 3D spectra from just ~1.5 mg of uniformly labeled amyloid fibrils packed in a standard 3.2 mm rotor. As a proof of concept, we apply this approach to RIPK3, a key protein in immune signaling that forms functional amyloid assemblies. Using standard 3D experiments (NCACX and NCOCX), we obtain ^13^C and ^15^N backbone assignments and secondary structure information, despite the limited sample quantity and the use of only moderate magnetic fields. These findings highlight the potential of CPMAS CryoProbes to shift the paradigm in mass-limited SSNMR studies, from relying exclusively on ^1^H-detection and fast MAS to reembracing ^13^C-detected strategies.

## Introduction

Solid-state nuclear magnetic resonance (SSNMR) spectroscopy has emerged as a powerful tool to investigate the structure of biomolecular assemblies such as amyloid fibrils, membrane proteins, and phase-separated condensates, which are often inaccessible to solution-state techniques due to their size, heterogeneity, or insolubility [1–4]. However, a major limitation of SSNMR, particularly when using ^13^C detection, is its intrinsic low sensitivity, which typically necessitates several milligrams of isotopically labeled protein to obtain high-resolution multidimensional spectra. This requirement poses a significant challenge when the sample is precious, difficult to express, or aggregates during preparation, as is often the case for functional or pathological amyloids.

In recent years, significant progress in hardware development, most notably the introduction of fast magic-angle spinning (MAS) rotors and ^1^H-detected pulse sequences, has enabled SSNMR studies on low milligram to submilligram quantities of biomolecules [5–7]. These approaches typically rely on reduced-diameter rotors (e.g., 0.7–1.3 mm), where enhanced sensitivity arises from ^1^H detection under fast spinning conditions (60–110 kHz MAS) [8–11]. More recently, 0.4 mm rotors capable of spinning up to 160 kHz have been developed, further increasing sensitivity and resolution in proton-detected experiments [12]. While highly effective in boosting sensitivity, these strategies introduce challenges such as limited chemical shift dispersion in the proton dimension that often requires this advanced probe technology to be combined with high or ultra-high field magnets not yet available in all laboratories, restricting its accessibility.

An alternative and highly complementary solution to the sensitivity bottleneck in SSNMR is the use of cryogenically cooled CPMAS probes, which enhance the signal-to-noise ratio by cooling the electronics while maintaining the sample at a regulated temperature. In a landmark study, Hassan et al. demonstrated the full potential of this technology for structural biology by acquiring high-quality 3D ^13^C-detected spectra of amyloid fibrils packed into a 3.2 mm rotor using several milligrams of protein within a single night [13]. Their results showcased the capability of the CPMAS CryoProbe to dramatically reduce acquisition times for complex multidimensional experiments when sample availability is not a constraint. In the present work, the opposite regime is explored, demonstrating that this cryogenic hardware can be leveraged to enable ^13^C-detected assignments of mass-limited biological samples in the 1–2 mg range and at moderate fields (14.1 T) quantities that would normally only be considered for ^1^H-detected fast MAS approaches and at high to ultra-high fields.

As a test case, we selected the RHIM (RIP homotypic interaction motif) region of RIPK3, a protein that plays a central role in necroptotic cell death and immune signaling [14]. The RHIM of RIPK3 forms highly ordered amyloid fibrils upon self-assembly that mediate functional signaling complexes [15], serving as a reference system for studying amyloid structure and specificity. Its biological relevance, well-characterized aggregation behavior, and previously solved structure [15] make it an ideal benchmark to test the capabilities of the CPMAS CryoProbe under mass-limited conditions. Using only ~1.5 mg of uniformly labeled RIPK3 fibrils packed in a 3.2 mm rotor, we acquired a full set of 2D and 3D ^13^C-detected spectra that enables backbone assignment, chemical shift-based secondary structure analysis, and identification of long-range contacts consistent with the known fold.

This study establishes that ^13^C-detected multidimensional SSNMR experiments can be successfully performed on biologically relevant protein assemblies available in limited amounts using CPMAS CryoProbes and moderate magnetic fields (14.1 T). This approach preserves the benefits of ^13^C chemical shift dispersion and avoids the hardware demands of ^1^H detection via fast MAS and high to ultra-high magnetic fields, while remaining compatible with standard pulse sequences. By exploiting the sensitivity gains of the CPMAS CryoProbe, we show that structure determination by conventional ^13^C-based experiments is feasible even in scenarios where material scarcity would typically preclude such analysis and using physiological sample temperatures. Our results position this technology as a practical and robust alternative to fast MAS for structural studies of mass-limited biomolecular assemblies, particularly in contexts where ^13^C chemical shift resolution is advantageous.

## Materials and Methods

### Protein expression and fibril preparation

The RHIM domain of human RIPK3 (residues 387–518) was expressed in Escherichia coli BL21(DE3) cells using a pET-11a vector. Uniform ^13^C,^15^N labeling was achieved by growing cells in M9 minimal medium supplemented with ^13^C-glucose and ^15^NH_4_Cl as sole carbon and nitrogen sources, respectively. After induction with IPTG (1 mM, 18 h at 37°C), the protein accumulated in inclusion bodies, which were harvested, washed, and solubilized in 6 M guanidinium chloride. Purification was performed under denaturing conditions using Ni^2+^-NTA affinity chromatography, during which the protein was exchanged to 8 M urea, followed by fibril formation through dialysis against assembly buffer (20 mM Tris, pH 7.4) for three days at room temperature. Fibrils were pelleted by centrifugation (10,000 ×g, 1 h), washed to remove residual monomers, and transferred into a 3.2 mm rotor using a home-built set of tools described in Gelardo and Titaux-Delgado [16], specifically designed to guide the material directly into the rotor by centrifugation (20,000 x g for 30 min). This procedure uses standard 1.5 mL Eppendorf tubes and conventional benchtop centrifuges.

### Solid-state NMR experiments

All SSNMR experiments were performed on a 600 MHz (14.1 T) Bruker Avance NEO spectrometer equipped with a 3.2 mm HCN CPMAS CryoProbe operating at a MAS rate of 10 kHz. Approximately, 1.5 mg of uniformly ^13^C,^15^N-labeled RIPK3 fibrils were used for all the experiments. The temperature was regulated using a Bruker BCU-II cooling unit. Multidimensional ^13^C-detected experiments shown in this work included 2D NCA [17], 3D NCACX, and 3D NCOCX [18]. The two 3D experiments were acquired with 10% non-uniform sampling (NUS). Cross polarization (CP) was used for ^1^H-^15^N and ^15^N-^13^C transfers, with ramped CP on the proton and carbon channels, respectively. CP contact times were 1.5 ms (^1^H–^15^N) and 5 ms (^15^N–^13^C). 100 kHz SPINAL-64 decoupling was applied during double CP and during acquisition. Acquisition parameters are summarized in Table 1.

### Data processing and analysis

All NMR data were processed using Bruker TopSpin 4.4.1 and analyzed with Poky [19]. Spectra were zero-filled and apodized with a sine bell function prior to Fourier transformation. Chemical shift referencing was performed using the downfield peak of adamantane as an external standard. Secondary structure was assessed by analyzing Δ(^13^Cα−^13^Cβ) secondary shifts, using the Poulsen dataset for disordered proteins [20,21].

## Results and Discussion

### High-quality ^13^C-detected spectra from ~1.5 mg of amyloid fibrils using an HCN CPMAS CryoProbe

**Figure 1:**
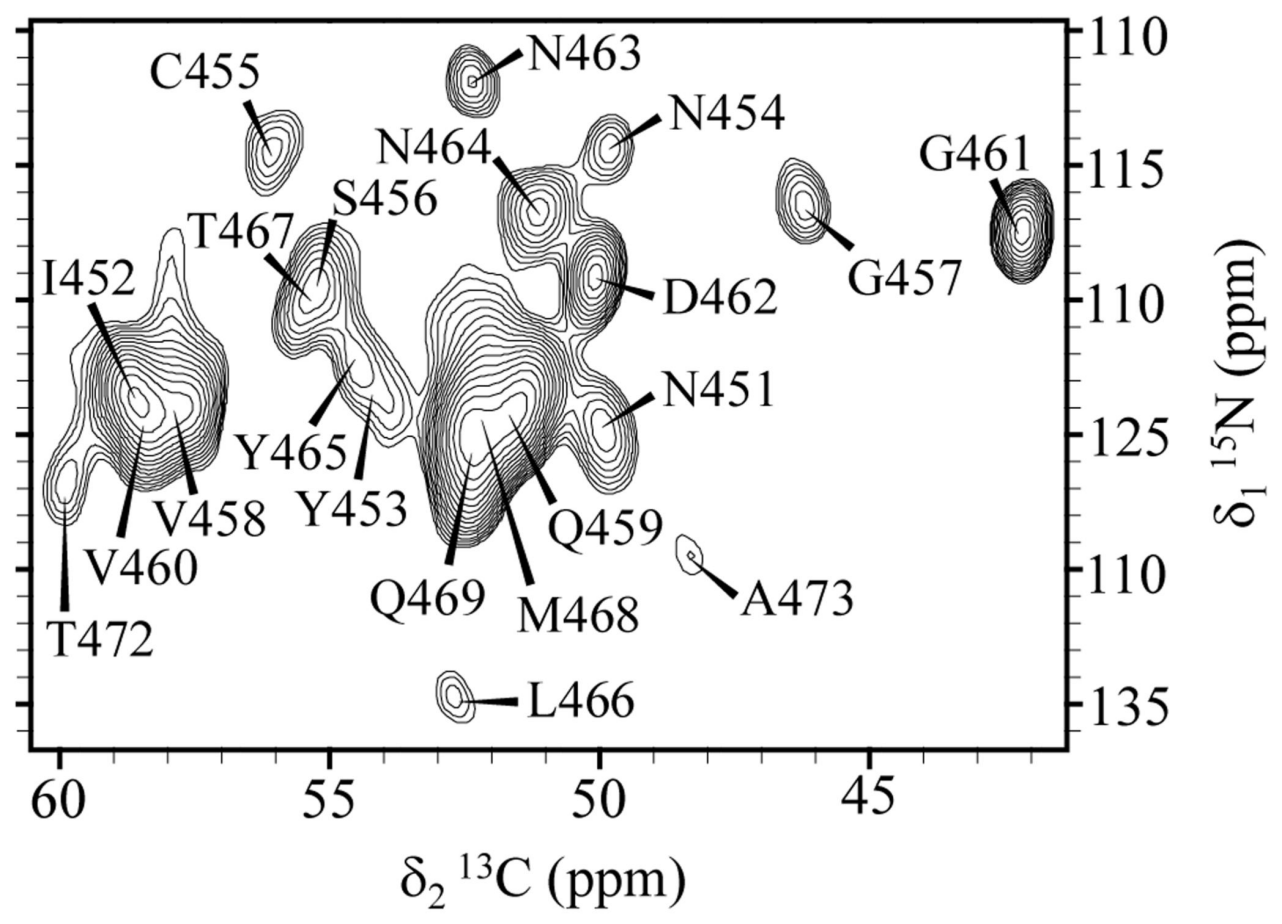
Assigned 2D ^13^C-^15^N correlation spectrum of RIPK3 amyloid fibrils acquired with a CPMAS CryoProbe. 2D NCA spectrum recorded from ~1.5 mg of uniformly labeled RIPK3 RHIM fibrils packed in a 3.2 mm rotor using an HCN CPMAS CryoProbe. Assignments are taken from [15]. The spectrum was acquired at 600 MHz and at 37 ºC (sample temperature).

To evaluate the potential of HCN CPMAS CryoProbe technology for structural studies on mass-limited biological samples, we first recorded a 2D ^13^C-^15^N correlation spectrum (NCA) of RIPK3 RHIM fibrils using ~1.5 mg of sample packed into a 3.2 mm rotor and centered with Teflon spacers. Despite the low sample quantity, the spectrum displayed excellent signal-to-noise and chemical shift resolution (Figure 1), enabling the identification and assignment of individual spin systems in the fibril core.

The spectral quality observed is comparable to that typically obtained from fully filled 3.2 mm rotors of sample under conventional conditions [15]. Notably, the acquisition time for this experiment was under 12 hours, highlighting the sensitivity gains provided by cryogenic detection.

The chemical shift dispersion observed in the ^13^Cα and ^15^N dimensions facilitated the discrimination of backbone resonances in the amyloid core, setting the stage for multidimensional experiments that are often required for full assignment.

**Figure 2:**
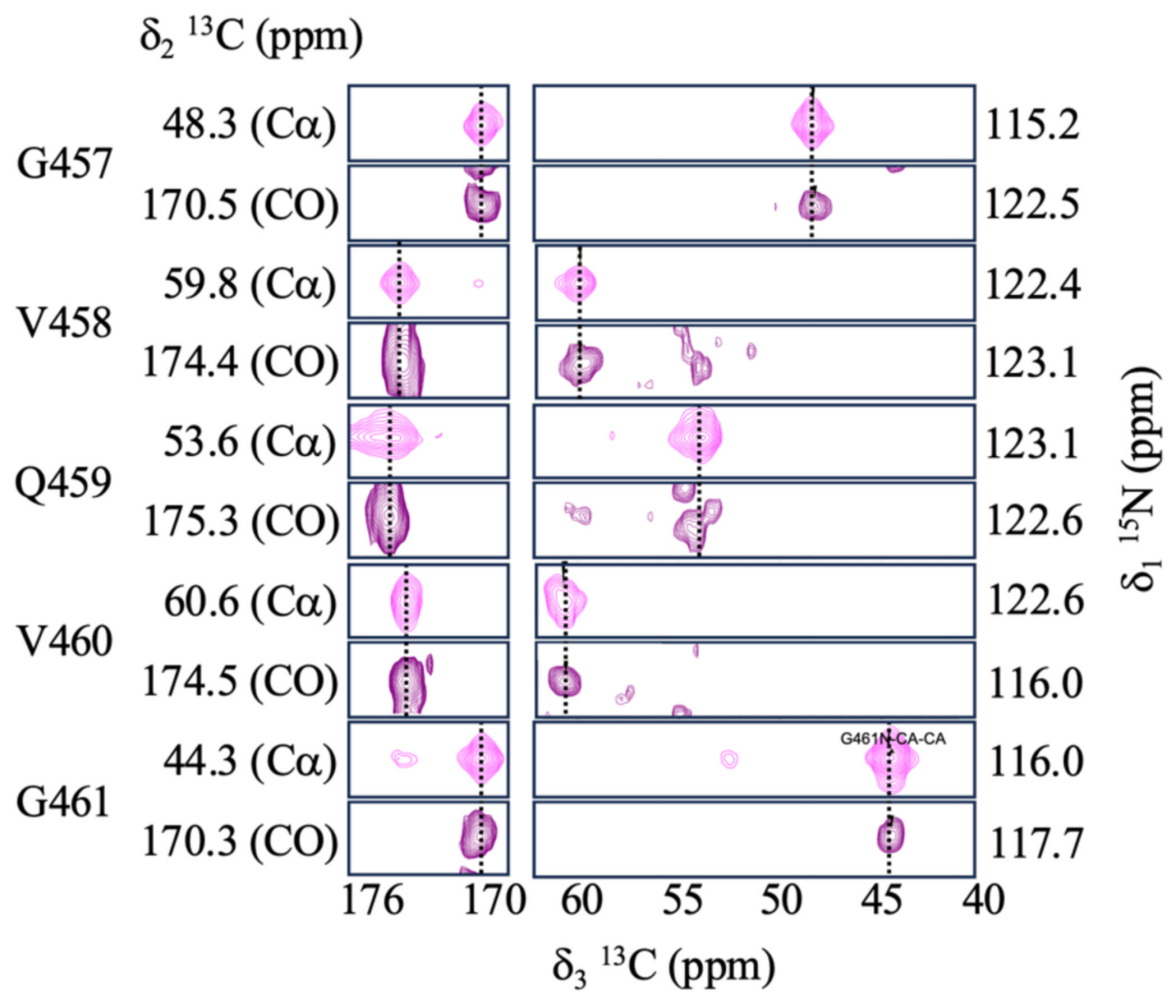
Strip plots from 3D experiments showing backbone walk of RIPK3 fibrils. Representative strips from NCACX and NCOCX 3D experiments illustrating sequential correlations that support backbone assignments. Assignments are taken from [15].

Building on the quality of the 2D NCA spectrum, we proceeded to acquire standard 3D ^13^C-detected experiments, namely NCACX and NCOCX, to correlate sequential backbone ncueli within the amyloid core of RIPK3 RHIM fibrils. All experiments were performed on the same ~1.5 mg sample, at 600 MHz and 37 ºC, using conventional CP-based transfer schemes. The temperature was chosen to approach the physiological range at which RIPK3 forms functional amyloids in the cell. Representative strip plots are shown in Figure 2, illustrating the sequential walk along the protein backbone. The correlations were well resolved and exhibited sufficient sensitivity to allow unambiguous connectivity between neighboring residues. The successful execution of these 3D experiments under such stringent mass constraints demonstrates the feasibility of using CPMAS CryoProbes for full backbone assignments in amyloid fibrils. Importantly, the ability to perform conventional ^13^C-based assignment strategies preserves the advantages of chemical shift dispersion and minimizes spectral overlap, especially valuable in β-sheet-rich systems such as amyloids.

### Chemical shift analysis confirms β-sheet structure in the RIPK3 amyloid core

**Figure 3:**
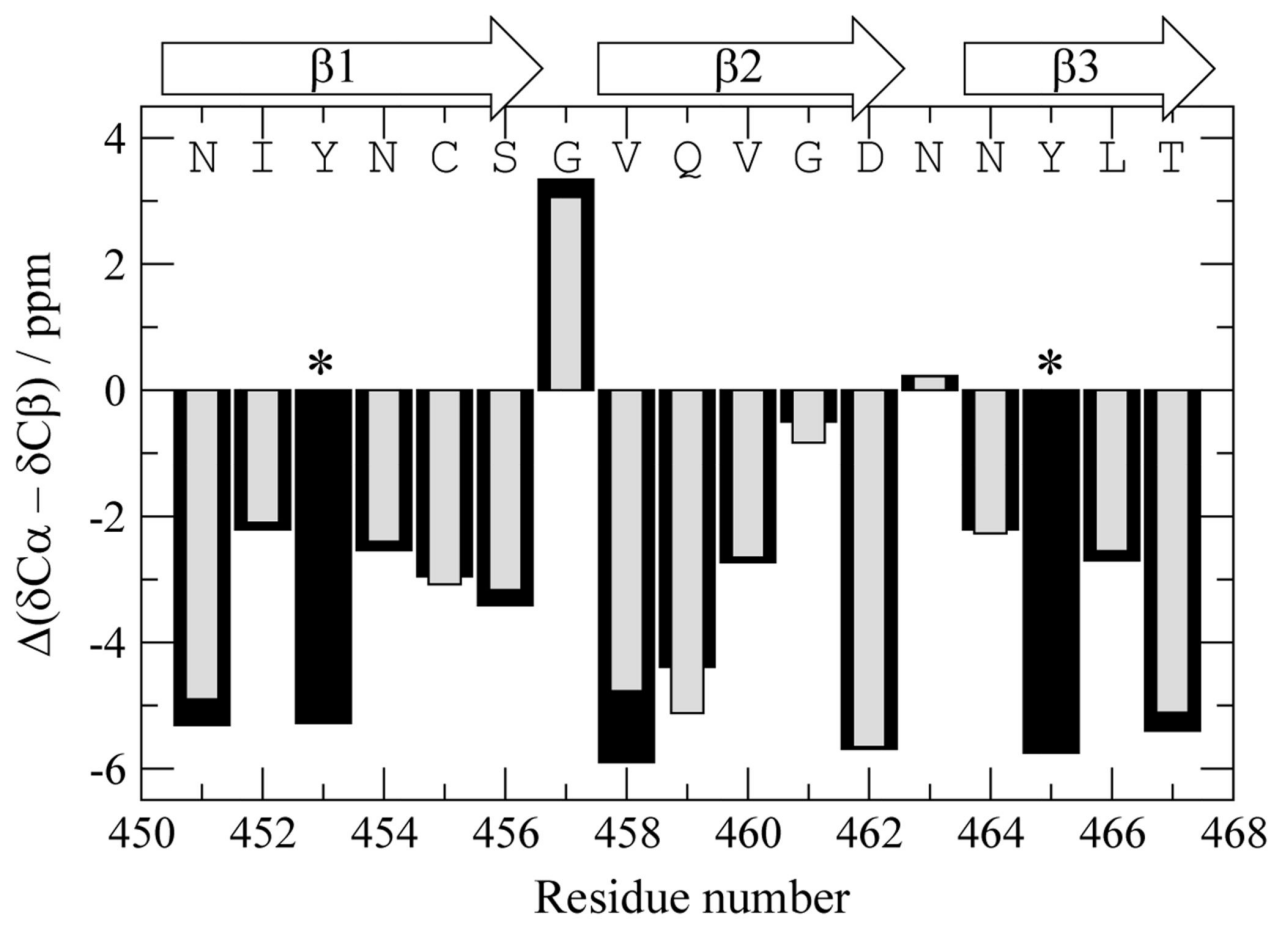
Secondary structure of RIPK3 RHIM fibrils derived from chemical shift analysis. Plot of Δ(δ^13^Cα− δ^13^Cβ) secondary chemical shifts for assigned residues in the sample from this study (~1.5 mg; black bars) and from previously published data [15] (grey bar), indicating three regions of β-sheet structure. The data confirm the presence of an extended β-strand core typical of RHIM amyloids. Residues marked with two asterisks (**) correspond to positions where ^13^Cβ assignments were not reported in the reference dataset by Wu et al., [15] but were assigned in this study and are consistent with the three-stranded amyloid core.

To assess the secondary structure of the RIPK3 RHIM fibrils obtained from our ~1.5 mg sample, we compared our chemical shift data to previously reported backbone assignments on the same system [15]. Specifically, we analyzed the difference between ^13^Cα and ^13^Cβ secondary shifts (ΔCα−Cβ), a well-established indicator of secondary structure in solid-state NMR, and plotted them alongside the published values (Figure 3).

The majority of assigned residues display strongly negative ΔCα−Cβ values, consistent with an extended β-sheet conformation, in excellent agreement with the published amyloid structure of RIPK3, where the RHIM forms a parallel in-register β-sheet core [15]. The high level of agreement validates both the quality of the data acquired with the CPMAS CryoProbe and the robustness of ^13^C-detected SSNMR experiments for structural analysis under limiting conditions. Moreover, the well-defined nature of the shift patterns supports the notion that RIPK3 forms a homogeneous and ordered amyloid assembly, reinforcing its utility as a benchmark system for SSNMR method development.

## Conclusions

This study demonstrates that high-resolution ^13^C-detected solid-state NMR spectra, including complete 3D assignment datasets, can be acquired from as little as 1-1.5 mg of uniformly labeled protein using a triple resonance CPMAS CryoProbe. By applying this approach to the well-characterized amyloid fibrils formed by the RHIM domain of RIPK3, we show that backbone correlations and secondary structure features can be obtained without resorting to fast MAS or ^1^H detection, and with results consistent with those obtained from larger sample amounts and at higher magnetic fields.

These findings establish cryogenically cooled CPMAS probes as powerful tools for obtaining structural information on biomolecular assemblies under mass-limited conditions. By enabling ^13^C-detected experiments at moderate magnetic fields, this approach complements ^1^H-detection by preserving the chemical shift dispersion and robustness of ^13^C-based strategies, which is particularly valuable when studying precious, aggregation-prone, or otherwise difficult-to-produce protein systems.

## Figures and Tables

**Table 1:** Acquisition parameters for ^13^C-detected experiments on ~1.5 mg hydrated RIPK3 fibrils using a 3.2 mm CPMAS CryoProbe.

Experiment	DARR Mixing time (ms)	Acquisition time (ms)	Spectral widths (ppm)	Recycle delay (s)	Number of scans	Time per increment	Total acquisition time
NCACX	20	11.5 (^13^C) 6.0 (^15^N)	330 (^13^C, f3) 66 (^13^C, f2) 88 (^15^N, f1)	1.5	128	~1.5 min	~2 days
NCOCX	20	11.5 (^13^C) 6.0 (^15^N)	330 (^13^C, f3) 66 (^13^C, f2) 88 (^15^N, f1)	1.5	128	~1.5 min	~2 days
NCA	-	11.5 (^13^C) 6.0 (^15^N)	293 (^13^C, f2) 66 (^15^N, f1)	2.0	128	~1 min	3 h
